# Time-dependent prognostic accuracy measures for recurrent event data

**DOI:** 10.1093/biomtc/ujae150

**Published:** 2024-12-26

**Authors:** R Dey, D E Schaubel, J A Hanley, P Saha-Chaudhuri

**Affiliations:** Department of Epidemiology, Biostatistics and Occupational Health, McGill University, Montreal, QC H3A 0G3, Canada; Department of Biostatistics, Epidemiology and Informatics, University of Pennsylvania Perelman School of Medicine, Philadelphia, PA 19104-6021, United States; Department of Epidemiology, Biostatistics and Occupational Health, McGill University, Montreal, QC H3A 0G3, Canada; Evidence Generation and Advanced Analytics Biogen Digital Health, Biogen, Cambridge, MA 02142, United States

**Keywords:** prognostic accuracy, recurrent events, semiparametric model, sparse, time-dependent

## Abstract

In many clinical contexts, the event of interest could occur multiple times for the same patient. Considerable advancement has been made on developing recurrent event models based on or that use biomarker information. However, less attention has been given to evaluating the prognostic accuracy of a biomarker or a composite score obtained from a fitted recurrent event-rate model. In this manuscript, we propose novel measures to characterize the prognostic accuracy of a marker measured at baseline in the presence of recurrent events. The proposed estimators are based on a semiparametric frailty model that accounts for the informativeness of a marker and unobserved heterogeneity among patients with respect to the rate of event occurrence. We investigate the asymptotic properties of the proposed accuracy estimators and demonstrate these estimators’ finite sample performance through simulation studies. The proposed estimators have minimal bias and appropriate coverage. The estimators are applied to evaluate the performance of a baseline forced expiratory volume, a measure of lung capacity, for repeated episodes of pulmonary exacerbations in patients with cystic fibrosis.

## 1 INTRODUCTION

Recurrent events are common in many clinical or epidemiological contexts when a subject or patient repeatedly experiences an event of interest. Examples include asthma attacks, epileptic seizures, and bone fractures (Cook and Lawless, 2007). We focus on recurrent event data obtained from a prospective study in which the subjects are recruited and the marker is measured at baseline. Subjects are followed up over time, and during follow-up, a subject can repeatedly experience the same type of event. The follow-up can be terminated either by the loss to follow-up or by the end of the study. Consider, for example, a study of patients with cystic fibrosis (CF). The patients with CF have diminished lung capacity and experience repeated pulmonary exacerbations characterized by an increased shortness of breath, hemoptysis, oxygen desaturation, and decreased lung capacity. Eventually, 80%-95% of CF patients succumb to respiratory failure brought on by pulmonary exacerbations (Lyczak et al., 2002). The forced expiratory volume (FEV), a measure of lung capacity, is an important marker of the risk of future exacerbation, with lower FEV more indicative of a higher risk of exacerbation. Identifying patients at high risk will help the clinician determine medical management or the choice and timing of interventions.

Statistical evaluation of the prognostic accuracy of a marker is essential. We focus on a scalar marker, a single biomarker such as FEV or a scalar composite score, for example, the CF-ABLE score (McCarthy et al., 2013) or CF clinical score (Kanga et al., 1999). A composite score can be derived as a linear combination of a set of estimated coefficients from the fitted regression model and risk factors (Hansen, 2008). A marker’s prognostic performance refers to (1) the discriminative ability of a marker, that is, how well it separates patients with a higher number of events from patients with a lower number of events and (2) its ability to quantify a patient’s risk of the recurrent episodes given the value of the marker.

Over the last decade, there have been significant advances in statistical modeling of recurrent events data (eg, Andersen and Gill, 1982; Lin et al., 2000; Schaubel et al., 2006). However, less attention has been given to assessing the accuracy of a fitted recurrent event-rate model. In this manuscript, we propose a new method for characterizing the prognostic accuracy of a marker in the presence of recurrent events. The receiver operating characteristic (ROC) paradigm is a convenient and useful tool for assessing the accuracy of a *diagnostic* model for a binary outcome. In principle, the concept of diagnostic accuracy (Pepe, 2003) extends naturally to the patient’s “future” disease state. However, for follow-up studies with survival outcomes, the “disease” or the outcome status of the patients can vary over time, and not all patients are followed for the same length of time (eg, Dey et al., 2020). Time-dependent true-positive fraction (TPF), false-positive fraction (FPF), and ROC measures have been developed for evaluating a marker’s *prognostic* performance using censored data with a single endpoint (eg, Heagerty et al., 2000; Heagerty and Zheng, 2005; Dey et al., 2023). These measures could be used directly in recurrent data settings if we were to terminate the follow-up for a patient when the first event occurs or (if it has not yet) when the study ends. Typically, when evaluating the predictive ability of a marker in the presence of recurrent events, many analyses (eg, Sultan et al., 2015; Cabral et al., 2020) ignore the inherent correlation within recurrent events or limit themselves to the use time-to-first event. For instance, the research presented in Sultan et al. (2015), a conventional Cox proportional hazards model was employed to predict the risk of recurrent strokes in individuals with Moyamoya arteriopathy. Based on this, the area under the ROC was reported for predicting recurrent stroke. This analysis ignored the inherent correlation within recurrent events. By not accounting for the correlation between repeated events within each patient, a significant degree of precision may be sacrificed. Failure to incorporate the correlation between subsequent events in the analysis could lead to suboptimal predictive accuracy (Kim et al., 2018).

We were only able to find one study of accuracy measures to evaluate the accuracy of a marker or a score derived from a recurrent event model (Kim et al., 2018). This motivated us to propose novel measures to characterize the prognostic accuracy of a marker when the event-of-interest is recurrent. In this manuscript, we propose prognostic accuracy measures applicable to recurrent event data.

We do so by developing semiparametric estimators for making inferences about the prognostic accuracy measures that accommodate recurrent events. A standard assumption in the recurrent event literature is that event counts follow a Poisson process (eg, Cook and Lawless, 2007; Kalbfleisch and Prentice, 2011). The informativeness of the marker can be incorporated in a Poisson process by specifying the intensity as a function of follow-up time $t$ and the marker(s). A model is perfectly specified when all possible relevant markers are accounted for. However, typically, not all markers that influence the distribution of the event time are included in a model. Unavoidably, there is unobserved heterogeneity. One way of accounting for this unobserved heterogeneity is to include random effects (frailties) in the model (Duchateau and Janssen, 2007). Our proposed estimators of accuracy measures are based on a semiparametric frailty model (Klein, 1992) that accounts for the informativeness of the marker and unobserved heterogeneity among patients with respect to the rate of event occurrence.

We investigate the asymptotic properties of the proposed accuracy estimators and demonstrate the finite sample performance of these estimators through simulation studies. We then apply the proposed measures to evaluate the performance of FEV at enrollment for repeated episodes of pulmonary exacerbations in patients with CF.

The remainder of the manuscript is organized as follows. In Section 2.1, we provide the notation. In Sections 2.2 and 2.3.1, we propose prognostic accuracy measures and their estimation, respectively. We report simulation results in Section 4 and illustrate our methodology with a real-life example in Section 5. We conclude with a discussion in Section 6.

## 2 METHODS

Our proposed accuracy measures in the context of recurrent events will build on “diagnostic” and “prognostic” accuracy measures in the simple case. A marker’s discriminative ability is typically addressed with correct classification rates such as the TPF and FPF (Pepe, 2003). The ROC curve is a plot of the TPF against FPF across all decision thresholds, and the area under the ROC curve (AUC) provides a global summary of a marker’s performance (Hanley and McNeil, 1982).

However, TPF and FPF do not provide a direct measure of an individual patient’s risk. Clinicians and individual patients are most concerned with this latter quantity, given patient-specific characteristics (eg, markers). The positive predictive value (PPV) and negative predictive value (NPV) are critical in addressing this risk (Pepe, 2003).

Our objectives are 2-fold: (1) to propose meaningful prognostic accuracy measures to evaluate the prognostic performance of a baseline marker for recurrent events context while after taking right-censoring into account, (2) to propose semiparametric estimators of these measures.

To that end, we first introduce the notation in Section 2.1, propose the accuracy summaries and outline the estimation.

### 2.1 Notation

Let $M$ denote a scalar marker measured at baseline that could potentially be used in estimating the risk of recurrences. The number of events for a subject over the interval [0, $t$] is given by $N(t)$. Let $C$ denote the censoring time. Assume that $N(.)$ and $C$ are independent conditional on $M$. We have a random sample of $n$ independent individuals. Let $0 < t_{i1} < t_{i2} < \dots < t_{in_{i}}$ denote the event-times for the $i$th individual, where $t_{ij}$ is the time of the $j$th occurrence of the event $(j=1,2,\dots ,n_{i})$ in subject $i$. For simplicity, let $K=\textrm {max}\, (n_{1},n_{2},\dots ,n_{n})$ be the maximum number of recurrences within [0, $t$] and $K\ge 2$. We use $t^{-}$ to denote time that is infinitesimally smaller than $t$. In addition, we let $\Delta N_{i}(t) = N_{i}(t+\Delta t^{-})-N_{i}(t^{-})$ denote the number of events in the interval $[t, t+\Delta t)$, and let $H_{i}(t) = \lbrace N_{i}(s):0\le s < t\rbrace$ denote the history of the process up to time $t$. The observed event process is denoted as $N_{i}^{o}(t) = N_{i}(t \wedge C_{i})$ over the total observation window $[0, \tau ]$, where a $\wedge$  *b* = min(*a, b*). Let $m_{i}$ be the observed realization of $M_{i}$. Hence, the observed data will be $\lbrace m_{i}, C_{i}, N^{o}_{i}(t);0\le t \le \tau \ \rbrace (i=1,2,...,n).$

### 2.2 The proposed prognostic accuracy measures

In recurrent event time data, the outcome for each participant is characterized by a counting process that counts the total number of events observed within a given time period. We define 2 groups of patients (higher and lower risk) based on the total number of events $N(t)$ over time window $[0,t]$. The “high-risk” patients or “cases” are those who will experience at least “$r$” events over time window $[0,t]$, that is, $N(t)\ge r$, and the “low-risk” patients who will experience fewer than “$r$” events over the same time window, that is, $N(t)< r$. Here, $r$ is an arbitrary number that can range from 1 to $K$.

#### 2.2.1 TPF/FPF/ROC measures

Consider a threshold $c$ for the marker. Given the definition of high and low risk, we define the true positive probability associated with $M$ as the expected fraction of cases who have a marker score greater than $c$.


(1) \begin{eqnarray*}
\text{TPF}^{(r)}(c,t) =\textrm {Pr}(M> c \mid N(t)\ge r),\, \, r=1,2,3,...,K. \\
\end{eqnarray*}

Similarly, the false positive probability associated with $M$ is based on those patients with the lowest risk, that is, $N(t)< r$.


(2) \begin{eqnarray*}
\text{FPF}^{(r)}(c,t)=\textrm {Pr}(M> c \mid N(t)< r).
\end{eqnarray*}

Let $c_{p_{0}}$ be the threshold that yields a false positive probability of $p_{0}$, $\text{FPF}^{(r)}(c_{p_{0}},t)=p_{0}$. The ROC curve evaluated at $\text{FPF}^{(r)}(c_{p_{0}},t)=p_{0}$ is defined as $\text{ROC}^{(r)}(p_{0}, t)=\text{TPF}^{(r)}\big (c_{p_{0}}, t\big )$ for $p_{0}\, \, \epsilon \, \, [0,1]$. For a specific time-window $[0,t]$, it quantifies how well $M$ discriminates high-frequency patients from low-frequency patients. If the marker is useful for discriminating higher and lower risk patients, then the area under the $\text{ROC}^{(r)}(p_{0},t)$ curve, $\text{AUC}^{(r)}(t)$, will be higher than 0.5. For an uninformative marker, the $\text{ROC}^{(r)}(p_{0},t)$ curve will overlap with the diagonal line. Note that for $r=K=1$, the definitions of $\text{TPF}^{(r)}(c,t)$ and $\text{FPF}^{(r)}(c,t)$ correspond to the “cumulative” sensitivity and “dynamic” specificity at $t$ (Heagerty et al., 2000).

#### 2.2.2 PPV/NPV measures

The ROC curve does not provide a direct measure of an individual patient’s risk. Thus, to estimate the patient’s risk of experiencing recurrent events given the marker value, we extend the idea from the application to binary outcomes considered by Moskowitz and Pepe (2004) to recurrent event outcomes. First, we define the PPV for a fixed period of time $[0,t]$ as a plot of


(3) \begin{eqnarray*}
\text{PPV}^{(r)}(v,t)= \text{Pr}(N(t)\ge r\mid G(m)> v)
\end{eqnarray*}

versus $v$, for $v\, \, \, \epsilon \, (0,1).$ Here, $G(.)$ is the cumulative distribution function of $M$ in the population. The *x*-axis of a PPV curve displays the fraction of patients having “positive” marker value, when a “positive” is defined by exceeding the threshold corresponding to the $v$th percentile of $M$ in the population, that is, $G(m)> v$. The *y*-axis indicates the risk of having at least $r$ events over the time-interval $[0,t]$ among the subgroup of patients who have a positive marker value. The consideration of $v$ (marker quantile, rather than the raw marker value) in the *x*-axis of the PPV curve is appealing, since it provides a common scale for comparing multiple markers with raw values measured on different scales. To measure the predictive performance of a marker, in the PPV curve, we can add horizontal lines that correspond to the marginal probability of $N(t)\ge r$, that is, $\text{Pr}(N(t)\ge r)$. This serves as the benchmark PPV curve for a completely uninformative marker. Markers with PPV curves that rise more steeply and reach higher levels than horizontal lines can be deemed informative.

The PPV curve can be used for practical purposes due to its ease of interpretation and visualization of useful quantities. For example, if only patients in the top 10th percentile of markers are eligible for an intervention in practice, then we can observe the expected proportion of such patients who will experience at least $r$ events by time $t$, $\text{PPV}^{(r)}(.90,t)$. Conversely, if the aim is for a fraction $p$ to have at least $r$ events by time $t$, then we can observe the corresponding fraction $1 - v$ of the population that will be required positive with the marker, $[\hat{\text{PPV}}^{(r)}]^{-1}(p,t)=v$, from a monotonic PPV curve.

Similarly, the NPV of $M$ can be defined as


(4) \begin{eqnarray*}
\text{NPV}^{(r)}(v,t)=\text{Pr}(N(t)< r\mid G(m)\le v) .
\end{eqnarray*}

Unlike the PPV curve, the *x*-axis of a NPV curve displays the fraction of patients having “negative” result, that is, less than the threshold corresponding to the $v$th percentile of $M$ in the population, that is, $G(m)\le v$. The *y*-axis indicates the risk of having fewer than $r$ events over the time-interval $[0,t]$ among the subgroup of patients who have a “negative” value of the marker result.

### 2.3 Estimation

The proposed accuracy measures of interest can be rewritten as


(5) \begin{eqnarray*}
\text{TPF}^{(r)}(c,t)\, \, =\frac{\sum _{j=r}^{K}\int _c^\infty f_{j}(t,m)\, dG(m) }{\sum _{j=r}^{K} \int _{-\infty }^\infty f_{j}(t,m)\, dG(m) },
\end{eqnarray*}

(6) \begin{eqnarray*}
\text{FPF}^{(r)}(c,t)=\frac{\sum _{j=0}^{(r-1)}\int _c^\infty f_{j}(t,m)\, dG(m) }{\sum _{j=0}^{(r-1)} \int _{-\infty }^\infty f_{j}(t,m)\, dG(m) },
\end{eqnarray*}

where $f_{j}(t,m)=\textrm {Pr}(N(t)=j\mid M=m)$, $j=0,1,$$2,\dots ,K$. Similarly, we rewrite the expressions for the PPV and the NPV as


(7) \begin{eqnarray*}
\text{PPV}^{(r)}(v,t)=\frac{\sum _{j=r}^{K}\int _{G^{-1}(v)}^\infty f_{j}(t,m)\, dG(m) }{\text{Pr}(M> G^{-1}(v))},
\end{eqnarray*}

(8) \begin{eqnarray*}
\text{NPV}^{(r)}(v,t) =\frac{\sum _{j=0}^{(r-1)}\int _{-\infty }^{G^{-1}(v)} f_{j}(t,m) \, dG(m) }{\text{Pr}(M\le G^{-1}(v))}.
\end{eqnarray*}

where $v\, \epsilon \, [0,1].$ Note that $\text{Pr}(M\le G^{-1}(v))=$$G(G^{-1}(v))=v$.

To make inference about these accuracy measures, we first note that $G(m)$ can be estimated empirically. The other key quantity here is the conditional probability $f_{j}(t,m)$. Below, we describe the approach based on the semiparametric mixed Poisson model for estimating this quantity.

#### 2.3.1 Estimation with a semiparametric mixed Poisson model

The Semiparametric Mixed Poisson model (Cook and Lawless, 2007) is commonly used to analyze recurrent event data with inter-individual variation. To accommodate heterogeneity across individuals, this model incorporates a random effects (or frailty) component to a Poisson model, so that the conditional “subject-specific” intensity function is of the form


(9) \begin{eqnarray*}
\lambda (t\mid H_{i}(t),u_{i}) &=& \lim _{\Delta t \rightarrow 0} \frac{\textrm {Pr}(\Delta N_{i}(t)=1\mid H_{i}(t),u_{i})}{\Delta t} \\
&=& u_{i}\, \, \rho _{i}(t),
\end{eqnarray*}

where $u_{i}$ is an unobservable independent random effect for the $i$th individual $(i=1,2,...,n)$ and $\rho _{i}(t)$ is the rate function giving the marginal instantaneous probability of an event at time $t$. The terms $u_{1},u_{2},\dots , u_{n}$ are taken to be i.i.d. with distribution function $F_{1}(u)$, mean $\mathbb {E}(u_{i})=1$ and variance $\textrm {var}(u_{i})=\theta$. The parameter $\theta$ describes the degree of between-subject heterogeneity. Further, we assume that the rate function for $N_{i}(.)$ is $\rho _{i}(t)=\rho _{0}(t)\, \textrm {exp}(\beta \, m_{i})$, where $\rho _{0}(.)$ is the baseline rate function and $\beta$ is an unknown regression parameter. Given $u_{i}$ and $m_{i}$, the process $\lbrace N_{i}(t), t\ge 0\rbrace$ is Poisson with rate function $\rho _{i}(t)$. Further, we can write


(10) \begin{eqnarray*}
\mu _{i}(t)=\int _{0}^{t}\rho _{i}(s)ds=\mu _{0}(t)\, \text{exp}(\beta \, m_{i}),
\end{eqnarray*}

where $\mu _{0}(t)=\int _{0}^{t}\rho _{0}(s)ds$ is an unknown baseline mean function of the marginal recurrent event process. Then, given $m_{i}$ and $u_{i}$, the process $\lbrace N_{i}(t), t\ge 0\rbrace$ is Poisson with mean $u_{i}\, \mu _{i}(t)$.

In Equation (9), we assume $u_{i}$ has a Gamma distribution with mean 1, variance $\theta$, and density function


(11) \begin{eqnarray*}
\xi (u_{i};\theta )=\frac{u_{i}^{\theta ^{-1}-1}\, \textrm {exp}(-u_{i}/\theta )}{\theta ^{\theta ^{-1}}\, \Gamma (\theta ^{-1})}; \, \, \, u_{i}> 0.
\end{eqnarray*}

Given only $M_{i}=m_{i}$, the probability mass function, $f_{j}(t,m_{i})$, is then


(12) \begin{eqnarray*}
&&\textrm {Pr}(N_{i}(t)=j\mid M_{i}=m_{i})\\
&&\quad=\int _{0}^{\infty }\frac{\textrm {exp}[-u_{i}\, \mu _{i}(t)][u_{i}\, \mu _{i}(t)]^j}{j!} \xi (u_{i};\theta )\, du_{i},\\
&&\quad =\frac{\Gamma (j+\theta ^{-1})}{\Gamma (j+1)\, \Gamma (\theta ^{-1})}\, \, \frac{[\theta \, \mu _{i}(t)]^{j}}{[1+\theta \, \mu _{i}(t)]^{j+\theta ^{-1}}},
\end{eqnarray*}

where $j=0,1,2,\dots ,K.$ This is the form of the negative Binomial mass function.

Since the $u_{i}$’s are unobserved, the expectation-maximization algorithm is a natural estimation tool for obtaining the maximum likelihood estimates of the parameters $\Theta =\big (\beta , \theta , \mu _{0}(t)\big )$. The algorithm was proposed (Klein, 1992 and Nielsen et al., 1992), where in the E-step, the $u_{i}$’s are estimated and the likelihood is maximized in the M-step by substituting with the estimated $u_{i}$’s.

In this case, we consider the “complete data” likelihood, which would apply if the random effects were observed. The complete data log-likelihood is


\begin{eqnarray*}
\text{log}\, \, \textrm {L}_{\textrm {C}}(\Theta ) &=& \text{log}\, \, \textrm {L}_{\textrm {1}}(\beta , \mu _{0}(t))+\text{log}\, \, \textrm {L}_{\textrm {2}}(\beta , \mu _{0}(t))\\
&&+\,\text{log}\, \, \textrm {L}_{\textrm {3}}(\theta ),
\end{eqnarray*}

where


\begin{eqnarray*}
\text{log}\, \, \textrm {L}_{\textrm {1}}(\beta , \mu _{0}(t))=\sum _{i=1}^{n}\sum _{j=1}^{n_{i}}\Big [\text{log}\, \rho _{0}(t_{ij})-\text{log}\, \mu _{0}(\tau )\Big ],
\end{eqnarray*}

\begin{eqnarray*}
\text{log}\, \, \textrm {L}_{\textrm {2}}(\beta , \mu _{0}(t)) &=& \sum _{i=1}^{n}\Big [n_{i}\, (\text{log}\, u_{i}+\text{log}\, \mu _{0}(\tau )+\beta \, m_{i}) \\
&&-u_{i}\, \mu _{0}(\tau )\, \textrm {exp}(\beta \, m_{i})\Big ],
\end{eqnarray*}

\begin{eqnarray*}
\text{log}\, \, \textrm {L}_{\textrm {3}}(\theta ) &=& \sum _{i=1}^{n}\Big [(\theta ^{-1}-1)\, \text{log}\, u_{i}-u_{i}/\theta \\
&&-\text{log}\, \Gamma (\theta ^{-1})-\theta ^{-1}\, \text{log}\, \theta \Big ].
\end{eqnarray*}

The E-step at the $k$th iteration involves taking the expectation of the complete data log-likelihood with respect to $u_{i}$, but based on the conditional distribution of $u_{i}$ given $H_{i}(\tau )$, and evaluated at $\hat{\Theta }^{(k-1)}$, the parameter estimates from the previous iteration. The use of the Gamma frailty distribution is particularly appealing because $\xi (u_{i}\mid H_{i}(\tau ); \Theta )$ is also Gamma, with shape $\theta ^{-1} + N_{i}(\tau )$ and scale $\theta /(1 + \theta \, \mu _{i}(\tau ))$ and so the required expectations have a closed form. Specifically,


\begin{eqnarray*}
\mathbb {E}\lbrace u_{i}\mid H_{i}(\tau );\hat{\Theta }^{(k-1)}\rbrace =\frac{1+n_{i}\, \hat{\theta }^{(k-1)}}{1+\hat{\mu }_{i}^{(k-1)}\, \hat{\theta }^{(k-1)}}.
\end{eqnarray*}

At the M-step, we maximize $Q_{1}\big (\Theta ;\hat{\Theta }^{(k-1)}\big )+Q_{2}\big (\Theta ;\hat{\Theta }^{(k-1)}\big )+Q_{3}\big (\Theta ;\hat{\Theta }^{(k-1)}\big )$, where $Q_{a}\big (\Theta ;\hat{\Theta }^{(k-1)}\big )=\mathbb {E}\lbrace \text{log}\, \text{L}_{a}(.)\, \mid H_{i}(\tau );\hat{\Theta }^{(k-1)}\rbrace ,$  $a = 1,2,3.$ The iteration continues until convergence. To the end, we will have the estimate $\hat{\Theta }$ of $\Theta$. Now, the proposed accuracy measures of interest can be estimated as


\begin{eqnarray*}
\hat{ \text{TPF}}^{(r)}(c,t) &=& \frac{\sum _{j=r}^{K}\int _c^\infty \hat{f}_{j}(t,m)\, d\hat{G}(m) }{\sum _{j=r}^{K} \int _{-\infty }^\infty \hat{f}_{j}(t,m)\, d\hat{G}(m) }\\
&=& \frac{\sum _{i=1}^{n}\lbrace \sum _{j=r}^{K} \hat{f}_{j}(t,m_{i})\rbrace \, {\bf 1}(m_{i}> c) }{\sum _{i=1}^{n}\lbrace \sum _{j=r}^{K} \hat{f}_{j}(t,m_{i})\rbrace },
\end{eqnarray*}

\begin{eqnarray*}
\hat{\text{FPF}}^{(r)}(c,t) &=& \frac{\sum _{j=0}^{(r-1)}\int _c^\infty \hat{f}_{j}(t,m)\, d\hat{G}(m) }{\sum _{j=0}^{(r-1)} \int _{-\infty }^\infty \hat{f}_{j}(t,m)\, d\hat{G}(m) } \\
&=&\frac{\sum _{i=1}^{n}\lbrace \sum _{j=0}^{r-1} \hat{f}_{j}(t,m_{i})\rbrace \, {\bf 1}(m_{i}> c) }{\sum _{i=1}^{n}\lbrace \sum _{j=0}^{r-1} \hat{f}_{j}(t,m_{i})\rbrace },
\end{eqnarray*}

where $f_{j}(t,m_{i})$ is estimated from the Equation (12) as


\begin{eqnarray*}
\hat{f}_{j}(t,m_{i}) &=& \frac{\Gamma (j+\hat{\theta }^{-1})}{\Gamma (j-1)\, \, \Gamma (\hat{\theta }^{-1})}\, \, \frac{[\hat{\theta } \, \hat{\mu _{i}}(t)]^{j}}{[1+\hat{\theta } \, \hat{\mu _{i}}(t)]^{{j}+\hat{\theta }^{-1}}};\, \,\\
&&\quad j=0,1,2,\dots ,K,
\end{eqnarray*}

with $\hat{\mu }_{i}(t)= \hat{\mu }_{0}(t)\, \text{exp}(m_{i}\, \hat{\beta )}.$

The estimated ROC curve is defined as $\hat{\text{ROC}}^{(r)}(p_{0}, t)=\hat{\text{TPF}}^{(r)}\big (\lbrace \hat{\text{FPF}}^{(r)}\rbrace ^{-1}(p_{0}, t), t\big )$ for $p_{0}\, \, \epsilon \, \, [0,1]$. The area under this curve will be the estimated ${\text{AUC}}^{(r)}(t)$, $\hat{\text{AUC}}^{(r)}(t)$. Similarly, the PPV and NPV can be estimated as


(13) \begin{eqnarray*}
\hat{\text{PPV}}^{(r)}(v,t) &=& \frac{\sum _{j=r}^{K}\int _{\hat{G}^{-1}(v)}^\infty \hat{f}_{j}(t,m)\, d\hat{G}(m) }{1-v} \\
&=&\frac{\sum _{i=1}^{n}\lbrace \sum _{j=r}^{K} \hat{f}_{j}(t,m_{i})\rbrace \, {\bf 1}(m_{i}> \hat{G}^{-1}(v)) }{1-v},\\
\end{eqnarray*}

(14) \begin{eqnarray*}
\hat{\text{NPV}}^{(r)}(v,t) &=& \frac{\sum _{j=0}^{(r-1)}\int _{-\infty }^{\hat{G}^{-1}(v)} \hat{f}_{j}(t,m) \, d\hat{G}(m) }{v} \\
&=& \frac{\sum _{i=1}^{n}\lbrace \sum _{j=0}^{r-1} \hat{f}_{j}(t,m_{i}\rbrace \, {\bf 1}(m_{i}< \hat{G}^{-1}(v)) }{v},\\
\end{eqnarray*}

where $v\, \epsilon \, [0,1].$

## 3 INFERENCE

We first study the asymptotic properties of the proposed accuracy estimators. The key step in our theoretical development is the establishment of theory for the quantity ${f}_{j}(t,m)$. As shown in the Equation (12), the quantity ${f}_{j}(t,m)$ depends on $\Theta$. Let $\mathbb {M}$ be the support of the marker $M$.

Lemma 1 Given a fixed value of $K$, $j\, \, \epsilon \, \, (0,1,2,\dots ,K)$ and $t\, \, \epsilon \, \, [0,\tau ]$, $\sqrt{n}\, (\hat{f}_{j}(t,m)-{f}_{j}(t,m))$ converges to a mean zero Gaussian process on $\mathbb {M}$.

The proof is sketched in Appendix. Using this lemma, we can prove the following Theorems.

Theorem 1 Given a fixed value of $K$, $r\, \, \epsilon \, \, (2,\dots ,K)$ and $t\, \, \epsilon \, \, [0,\tau ]$, $\sqrt{n}\, (\hat{\text{TPF}}^{(r)}(c,t)-{\text{TPF}}^{(r)}(c,t))$, and $\sqrt{n}\, (\hat{\text{FPF}}^{(r)}(c,t)-{\text{FPF}}^{(r)}(c,t))$ converge to a mean zero Gaussian process on $\mathbb {M}$ with covariances in Equations (C.1) and (C.2), respectively.

The proof is sketched in Appendix.

Theorem 2 Given a fixed value of $K$, $r\, \, \epsilon \, \, (2,\dots ,K)$ and $t\, \, \epsilon \, \, [0,\tau ]$, $\sqrt{n}\, (\hat{\text{PPV}}^{(r)}(v,t)-{\text{PPV}}^{(r)}(v,t))$, and $\sqrt{n}\, (\hat{\text{NPV}}^{(r)}(v,t)-{\text{NPV}}^{(r)}(v,t))$ converge to a mean zero Gaussian processes on $\mathbb {M}$ with covariances based on the Equations (D.1) and (D.2), respectively.

The proof is sketched in Appendix.

The asymptotic properties of the ROC curves then follow from Theorem 1 by the functional delta method stated in Chapter 3.9 of van der Vaart and Wellner (1996).

Theorem 3 Given a fixed value of $K$, $r\, \, \epsilon \, \, (2,\dots ,K)$, and $t\, \epsilon \, \, \, [0,\tau ]$, $\sqrt{n}\, (\hat{\textrm {ROC}}^{(r)}(.;t)-{\textrm {ROC}}^{(r)}(.;t))$ converges to a mean zero Gaussian process on $\mathbb {M}$ with covariance in Equation (E.3).

The proof is sketched in Appendix.

The covariance formulas for these processes contain density functions. Smoothing techniques are needed to compute the standard errors (SEs) based on these formulas. We compute the SEs using the bootstrap method.

## 4 SIMULATION STUDY

We evaluated the finite sample performance of the proposed estimators through a series of simulation studies with varying degrees of unobserved heterogeneity of the recurrent event process. We conducted the simulations following the data generating framework designed in Jahn-Eimermacher et al. (2015). The recurrent event times for the $i$th individual $(i=1,2,\dots ,n)$ were generated from a proportional intensity model:


(15) \begin{eqnarray*}
u_{i}\, \Lambda _{0}(t)\, \text{exp}(\beta _{1} M_{i}),
\end{eqnarray*}

where $\Lambda _{0}(t)=\int _{0}^{t}\lambda _{0}(t)$ denotes the cumulative baseline hazard. Recall that $u_{i}$ denotes the random effect (frailty) that follows a Gamma distribution with $\mathbb {E}(u_{i}$) = 1 and var($u_{i}$) = $\theta$. The parameter $\theta$ describes the degree of between-subject heterogeneity.

We assumed $M_{i}$ follows a standard normal distribution. We considered 2 different sets of true values (0, 0.5) for $\beta _{1}$. For all simulation setups, we considered that $\theta$ varies from 0 to 1. As the value of $\theta$ increases, individuals tend to experience more events. To investigate the sensitivity to the assumption on the censoring mechanism, the censoring time $C_{i}$ was generated from the following scenarios: (1) complete follow-up: $C_{i}=\tau$ for all $i$; set $\tau$ = 5, and (2) independent censoring: $C_{i}$ was generated independently from Uniform$[0,\tau ]$, (3) covariate-dependent censoring: $C_{i}$ was generated from Uniform[1, $\tau$ + 1] if $M_{i} > 0$ and Exponential(1) + 1 if $M_{i} < 0$, and then truncated at $\tau$. The value of the cumulative baseline intensity $\Lambda _{0}(t)$ was fixed at 0.5 $t$ for all scenarios. The performance of the proposed methods was measured using bias, bootstrap SE, and coverage of the corresponding 90 percent confidence intervals based on 1000 simulated data sets consisting of sample size $n=500$. The standard deviation (SD) of the estimates over 1000 simulated data sets is used to assess the performance of the SE. We considered $r$ is fixed at 2 for all scenarios.

In addition, we investigated the performance of the estimators when the assumed Gamma frailty distribution in the semiparametric mixed Poisson model is misspecified. We generated the data using Equation (15) by assuming $u_{i}$ follows a Lognormal distribution with mean $\mathbb {E}(u_{i}$) = 1 and var($u_{i}$) = $\theta$. Then, we estimated the accuracy parameters using the proposed methods based on a Gamma frailty distribution.

Table 1 demonstrates the ROC measures of the marker, and Table 2 shows the PPV and NPV measures of estimating the risk of recurrences given the marker under correct frailty model specification. The estimators of the accuracy parameters showed negligible bias irrespective of the degree of unobserved heterogeneity $\theta$. For example, when the percentage of censoring is 10%, the bias of the estimate of AUC parameter for $\theta =0.5$ is −0.009. We also notice that the bias of the estimated values of AUC parameter strictly decreased as the frailty variance increased and the censoring percentage increased. The coverage probabilities are close to the nominal level of 90 percent except when $p_{0}$ is close to 0. For example, when the censoring is absent and $\theta =0.5$, the coverage probability of ROC for $p_{0}=0.1$ is 87%.

**TABLE 1:** Simulation results for the estimation of $\textrm {ROC}^{(r)}(p_{0},t)$ and $\textrm {AUC}^{(r)}(t)$ of a baseline marker as a function of probability of censoring, Pr(C); and the degree of heterogeneity, $\theta$. The $\textrm {ROC}^{(r)}(p_{0},t)$ curve is evaluated at a false positive probability of $p_{0}$. The value of $r$ is fixed at a value of 2. We consider $t=5$, which represents a study of length 5 time units. The $\textrm {ROC}^{(r)}(p_{0},t)$ and $\textrm {AUC}^{(r)}(t)$ estimations were conducted using the proposed semiparametric approach. The crude AUC($t$), cAUC, is estimated using the semi-parametric approach proposed in Heagerty et al. (2000) by terminating the follow-up for a patient when the first event occurs or (if it has not yet) when the study ends. The performance measures are bias (B), average of bootstrap standard errors (SEs), standard deviation (SD), coverage probability (CP) (nominal level is 0.9). To reduce the burden of notation, we have dropped the terms $r$ and $t$ from the notation of $\textrm {ROC}^{(r)}(p_{0},t)$ and $\textrm {AUC}^{(r)}(t)$. Here, the $\textrm {ROC}^{(r)}(p_{0},t)$ and $\textrm {AUC}^{(r)}(t)$ are represented by $\textrm {ROC}(p_{0})$ and $\textrm {AUC}$, respectively.

		$\theta$ = 0						$\theta$ = 0.5					$\theta$ = 1				
Censoring	Pr(C)	Estimator	True	B	SE	SD	CP	True	B	SE	SD	CP	True	B	SE	SD	CP
Complete follow-up	0%	$\textrm {ROC}(0.9)$	0.985	−0.002	0.0043	0.0038	0.92	0.975	0.001	0.002	0.008	0.87	0.969	0.001	0.002	0.009	0.90
		$\textrm {ROC}(0.5)$	0.840	−0.015	0.020	0.018	0.85	0.760	0.016	0.015	0.035	0.88	0.714	0.006	0.034	0.037	0.92
		$\textrm {ROC}(0.1)$	0.454	−0.030	0.044	0.041	0.86	0.287	0.028	0.024	0.057	0.88	0.231	0.008	0.042	0.040	0.88
		$\textrm {AUC}$	0.760	−0.009	0.015	0.014	0.88	0.691	0.012	0.011	0.024	0.87	0.652	0.004	0.022	0.020	0.90
		$\textrm {cAUC}$	0.760	−0.02				0.691	−0.031				0.652	−0.022			
Independent	10%	$\textrm {ROC}(0.9)$	0.981	−0.001	0.0041	0.003	0.92	0.975	0	0.005	0.004	0.91	0.967	0	0.009	0.008	0.91
		$\textrm {ROC}(0.5)$	0.820	−0.012	0.021	0.018	0.88	0.751	0	0.024	0.020	0.91	0.707	0.005	0.033	0.030	0.90
		$\textrm {ROC}(0.1)$	0.401	−0.028	0.044	0.040	0.86	0.270	0.001	0.035	0.031	0.89	0.225	0.008	0.039	0.035	0.91
		$\textrm {AUC}$	0.741	−0.011	0.015	0.013	0.88	0.682	−0.009	0.016	0.014	0.89	0.647	−0.004	0.021	0.019	0.89
		$\textrm {cAUC}$	0.741	−0.03				0.682	−0.017				0.647	−0.01			
	30%	$\textrm {ROC}(0.9)$	0.981	−0.002	0.0040	0.003	0.90	0.972	−0.001	0.005	0.004	0.91	0.965	−0.001	0.009	0.008	0.91
		$\textrm {ROC}(0.5)$	0.790	−0.014	0.021	0.019	0.86	0.734	−0.012	0.023	0.021	0.90	0.696	0.005	0.021	0.019	0.89
		$\textrm {ROC}(0.1)$	0.330	−0.020	0.040	0.038	0.87	0.250	−0.019	0.029	0.027	0.86	0.214	0.007	0.036	0.034	0.90
		$\textrm {AUC}$	0.712	−0.001	0.012	0.010	0.89	0.660	0.004	0.015	0.012	0.89	0.639	−0.002	0.021	0.019	0.89
		$\textrm {cAUC}$	0.712	−0.01				0.660	−0.026				0.639	−0.033			
Covariate-dependent	35%	$\textrm {ROC}(0.9)$	0.980	−0.002	0.004	0.004	0.90	0.973	−0.001	0.004	0.003	0.912	0.964	−0.001	0.008	0.007	0.913
		$\textrm {ROC}(0.5)$	0.780	−0.018	0.023	0.020	0.87	0.730	−0.013	0.025	0.022	0.90	0.690	0.003	0.023	0.002	0.89
		$\textrm {ROC}(0.1)$	0.330	−0.018	0.038	0.036	0.87	0.250	−0.021	0.030	0.032	0.87	0.214	0.004	0.034	0.033	0.90
		$\textrm {AUC}$	0.749	0.001	0.022	0.022	0.898	0.696	0.002	0.025	0.026	0.897	0.667	0.001	0.028	0.028	0.896
		$\textrm {cAUC}$	0.749	−0.03				0.696	−0.023				0.667	−0.031			

**TABLE 2:** Simulation results for the estimation of $\textrm {PPV}^{(r)}(v,t)$ and $\textrm {NPV}^{(r)}(v,t)$ of a baseline marker as a function of probability of censoring, Pr(C); and the degree of heterogeneity, $\theta$. The value of $r$ is fixed at a value of 2. We consider $t=5$, which represents a study of length 5 time units. The $\textrm {PPV}^{(r)}(v,t)$ and $\textrm {NPV}^{(r)}(v,t)$ estimations were conducted using the proposed semiparametric approach. The performance measures are bias (B), average of bootstrap standard errors (SEs), standard deviation (SD), coverage probability (CP) (nominal level is 0.9). To reduce the burden of notation, we have dropped the terms $r$ and $t$ from the notation of $\textrm {PPV}^{(r)}(v,t)$ and $\textrm {NPV}^{(r)}(v,t)$. Here, the $\textrm {PPV}^{(r)}(v,t)$ and $\textrm {NPV}^{(r)}(v,t)$ are represented by $\textrm {PPV}(v)$ and $\textrm {NPV}(v)$, respectively.

		$\theta$ = 0						$\theta$ = 0.5					$\theta$ = 1				
Censoring	Pr(C)	Estimator	True	B	SE	SD	CP	True	B	SE	SD	CP	True	B	SE	SD	CP
Complete follow-up	0%	$\textrm {PPV}(0.1)$	0.780	0.008	0.017	0.016	0.87	0.640	0.004	0.018	0.017	0.88	0.560	0.002	0.018	0.017	0.88
		$\textrm {PPV}(0.5)$	0.825	0.007	0.018	0.015	0.87	0.687	0.005	0.019	0.018	0.87	0.605	0.002	0.019	0.016	0.88
		$\textrm {PPV}(0.9)$	0.928	0.011	0.017	0.017	0.86	0.793	0.010	0.019	0.018	0.85	0.701	0.007	0.021	0.020	0.86
		$\textrm {NPV}(0.1)$	0.680	0.012	0.038	0.034	0.89	0.731	−0.002	0.036	0.033	0.84	0.734	−0.001	0.030	0.027	0.92
		$\textrm {NPV}(0.5)$	0.540	0.015	0.034	0.028	0.86	0.602	−0.003	0.029	0.026	0.82	0.632	−0.007	0.025	0.023	0.91
		$\textrm {NPV}(0.9)$	0.340	0.015	0.031	0.027	0.87	0.433	−0.005	0.021	0.019	0.88	0.495	−0.002	0.018	0.017	0.88
Independent	10%	$\textrm {PPV}(0.1)$	0.779	0.013	0.016	0.014	0.85	0.640	0.006	0.018	0.017	0.86	0.560	0.002	0.018	0.016	0.85
		$\textrm {PPV}(0.5)$	0.830	0.009	0.017	0.017	0.88	0.690	0.005	0.019	0.018	0.87	0.607	0.003	0.019	0.017	0.87
		$\textrm {PPV}(0.9)$	0.935	0.020	0.018	0.016	0.88	0.797	0.009	0.020	0.018	0.88	0.707	0.005	0.022	0.021	0.89
		$\textrm {NPV}(0.1)$	0.650	0.021	0.028	0.025	0.86	0.730	−0.009	0.035	0.032	0.85	0.732	−0.003	0.029	0.027	0.87
		$\textrm {NPV}(0.5)$	0.536	0.015	0.020	0.018	0.88	0.600	−0.008	0.028	0.026	0.86	0.630	−0.004	0.023	0.021	0.88
		$\textrm {NPV}(0.9)$	0.331	0.018	0.030	0.027	0.85	0.425	−0.007	0.021	0.019	0.88	0.493	−0.009	0.018	0.017	0.89
	30%	$\textrm {PPV}(0.1)$	0.778	0.011	0.014	0.013	0.86	0.640	0.005	0.019	0.017	0.88	0.559	0.002	0.019	0.018	0.84
		$\textrm {PPV}(0.5)$	0.832	0.008	0.019	0.018	0.88	0.692	0.006	0.020	0.019	0.88	0.612	0.003	0.020	0.018	0.89
		$\textrm {PPV}(0.9)$	0.937	0.022	0.019	0.017	0.87	0.804	0.004	0.019	0.017	0.85	0.713	0.002	0.023	0.021	0.89
		$\textrm {NPV}(0.1)$	0.647	0.023	0.029	0.027	0.87	0.725	−0.013	0.035	0.032	0.86	0.729	−0.003	0.029	0.026	0.84
		$\textrm {NPV}(0.5)$	0.534	0.013	0.021	0.019	0.88	0.593	−0.011	0.028	0.026	0.91	0.627	−0.004	0.023	0.021	0.88
		$\textrm {NPV}(0.9)$	0.323	0.020	0.029	0.027	0.86	0.420	−0.007	0.021	0.019	0.88	0.488	−0.006	0.019	0.018	0.89
Covariate-dependent	35%	$\textrm {PPV}(0.1)$	0.776	0.01	0.012	0.013	0.86	0.640	0.005	0.019	0.017	0.88	0.559	0.002	0.019	0.018	0.84
		$\textrm {PPV}(0.5)$	0.831	0.006	0.016	0.017	0.87	0.692	0.007	0.021	0.020	0.88	0.610	0.002	0.021	0.02	0.89
		$\textrm {PPV}(0.9)$	0.934	0.02	0.019	0.018	0.87	0.803	0.005	0.018	0.016	0.86	0.712	0.002	0.022	0.021	0.89
		$\textrm {NPV}(0.1)$	0.644	0.022	0.027	0.028	0.86	0.724	−0.012	0.033	0.032	0.87	0.73	−0.002	0.028	0.027	0.83
		$\textrm {NPV}(0.5)$	0.531	0.012	0.022	0.02	0.86	0.592	−0.01	0.027	0.026	0.92	0.626	−0.004	0.024	0.022	0.88
		$\textrm {NPV}(0.9)$	0.322	0.019	0.027	0.026	0.85	0.421	−0.006	0.02	0.019	0.87	0.485	−0.005	0.018	0.018	0.89

Further, we have compared the crude AUC (based on a time-to-first event analysis) and AUC estimates (based on a recurrent event analysis) to show the gain using our proposed approach over the crude AUC. As expected, the estimated value of crude AUC is smaller from the AUC estimates based on a recurrent event analysis irrespective of the degree of unobserved heterogeneity $\theta$.

We also investigated the finite sample behaviors of the estimators when the frailty distribution is misspecified. Under this scenario, recall that we generated the data by considering that the true frailty distribution is Lognormal and the marker is non-informative, that is $\beta _{1}=0$ in Equation (15). Hence, the expected ROC curve would lie diagonally on the null ROC curve. In turn, the true value of AUC will be 0.5. Tables 3 and 4 demonstrate that misspecifying the frailty distribution as Gamma (when it was actually Lognormal) does not cause much bias in the estimators of ROC, PPV, and NPV parameters irrespective of the degree of heterogeneity, $\theta$. For instance, in Table 3, when the magnitude of heterogeneity is moderate, that is $\theta =0.5$, all the estimated values of ROC and AUC parameters are about the same as the true values of the corresponding parameters. The estimates of the accuracy parameters are fairly close to their true values when $\theta$ equals one. In addition, the coverage probabilities are based on 90% estimated confidence intervals of the accuracy parameters close to the nominal value of 0.9. Additional simulations in Table 5 are considered to investigate the proposed estimators’ finite sample behavior by varying sample size and the values of $v$ in $\Lambda _{0}(t)=vt$ for scenario 1. The baseline hazard function varied across scenarios to demonstrate that similar areas under the ROC curve (AUC) can result from different censoring distributions. This approach highlights that different sample sizes can lead to similar measures of prognostic accuracy, such as the AUC, despite differences in the underlying mechanisms of censoring.

**TABLE 3:** Simulation results for estimation of $\textrm {ROC}^{(r)}(.)$ and $\textrm {AUC}^{(r)}(t)$ of a baseline marker when the assumed Gamma Frailty model misspecified. The value of $r$ is fixed at a value of 2. We assume the marker is non-informative. The $\textrm {ROC}^{(r)}(.)$ and $\textrm {AUC}^{(r)}(t)$ estimations were conducted using the proposed semiparametric approach. The crude AUC($t$), cAUC, is estimated using the semi-parametric approach proposed in Heagerty et al. (2000) by terminating the follow-up for a patient when the first event occurs or (if it has not yet) when the study ends. The performance measures are bias (B), an average of bootstrap standard errors (SEs), coverage probability (CP) (nominal level is 0.9). To reduce the burden of notation, we have dropped the terms $r$ and $t$ from the notation of $\textrm {ROC}^{(r)}(p_{0},t)$ and $\textrm {AUC}^{(r)}(t)$. Here, the $\textrm {ROC}^{(r)}(p_{0},t)$ and $\textrm {AUC}^{(r)}(t)$ are represented by $\textrm {ROC}(p_{0})$ and $\textrm {AUC}(t)$, respectively.

		$\theta = 0.5$						$\theta = 1$			
Censoring	Pr(C)	Estimator	True	B	SE	SD	CP	B	SE	SD	CP
Complete follow-up	0%	$\textrm {ROC}(0.9)$	0.90	−0.010	0.033	0.027	0.92	−0.008	0.028	0.024	0.90
		$\textrm {ROC}(0.5)$	0.50	0.000	0.054	0.049	0.91	−0.001	0.047	0.041	0.90
		$\textrm {ROC}(0.1)$	0.10	0.008	0.033	0.029	0.90	0.005	0.028	0.025	0.89
		$\textrm {AUC}$	0.50	−0.002	0.033	0.028	0.91	−0.001	0.028	0.024	0.88
		$\textrm {cAUC}$	0.50	−0.015				−0.012			
Independent	10%	$\textrm {ROC}(0.9)$	0.90	−0.008	0.031	0.029	0.92	−0.007	0.026	0.023	0.89
		$\textrm {ROC}(0.5)$	0.50	−0.002	0.05	0.046	0.90	0.000	0.044	0.041	0.89
		$\textrm {ROC}(0.1)$	0.10	0.007	0.031	0.027	0.91	0.006	0.027	0.024	0.91
		$\textrm {AUC}$	0.50	−0.002	0.03	0.027	0.89	0.000	0.027	0.024	0.89
		$\textrm {cAUC}$	0.50	−0.015				−0.010			
	30%	$\textrm {ROC}(0.9)$	0.90	−0.005	0.026	0.024	0.92	−0.006	0.024	0.023	0.91
		$\textrm {ROC}(0.5)$	0.50	−0.000	0.045	0.041	0.90	−0.002	0.041	0.038	0.90
		$\textrm {ROC}(0.1)$	0.10	0.005	0.026	0.023	0.90	0.004	0.024	0.022	0.92
		$\textrm {AUC}$	0.50	0.000	0.027	0.025	0.90	−0.002	0.025	0.023	0.90
		$\textrm {cAUC}$	0.50	−0.014				−0.011			
Covariate-dependent	35%	$\textrm {ROC}(0.9)$	0.90	−0.004	0.025	0.024	0.91	−0.004	0.025	0.023	0.91
		$\textrm {ROC}(0.5)$	0.50	−0.001	0.042	0.041	0.89	−0.001	0.039	0.038	0.90
		$\textrm {ROC}(0.1)$	0.10	0.003	0.024	0.023	0.90	0.003	0.023	0.022	0.91
		$\textrm {AUC}$	0.50	0.001	0.028	0.026	0.91	−0.001	0.024	0.022	0.91
		$\textrm {cAUC}$	0.50	−0.012				−0.010			

**TABLE 4:** Simulation results for the estimation of $\textrm {PPV}^{(r)}(v,t)$ and $\textrm {NPV}^{(r)}(v,t)$ of a baseline marker when the assumed Gamma Frailty model is misspecified. The value of $r$ is fixed at a value of 2. We assume the marker is uninformative. The $\textrm {PPV}^{(r)}(v,t)$ and $\textrm {NPV}^{(r)}(v,t)$ estimations were conducted using the proposed semiparametric approach. The performance measures are bias (B), an average of bootstrap standard errors (SEs), standard deviation (SD), coverage probability (CP) (nominal level is 0.9). To reduce the burden of notation, we have dropped the terms $r$ and $t$ from the notation of $\textrm {PPV}^{(r)}(v,t)$ and $\textrm {NPV}^{(r)}(v,t)$. Here, the $\textrm {PPV}^{(r)}(v,t)$ and $\textrm {NPV}^{(r)}(v,t)$ are represented by $\textrm {PPV}(v)$ and $\textrm {NPV}(v)$, respectively.

		$\theta = 0.5$						$\theta = 1$				
Censoring	Pr(C)	Estimator	True	B	SE	SD	CP	True	B	SE	SD	CP
Complete follow-up	0%	$\textrm {PPV}(0.1)$	0.583	−0.003	0.016	0.014	0.88	0.51	−0.003	0.020	0.018	0.91
		$\textrm {PPV}(0.5)$	0.583	−0.004	0.018	0.016	0.89	0.51	−0.003	0.030	0.028	0.90
		$\textrm {PPV}(0.9)$	0.583	−0.005	0.030	0.027	0.88	0.51	−0.003	0.02	0.017	0.92
		$\textrm {NPV}(0.1)$	0.417	0.002	0.031	0.029	0.87	0.49	0.003	0.030	0.027	0.88
		$\textrm {NPV}(0.5)$	0.417	0.002	0.021	0.019	0.88	0.49	0.003	0.021	0.018	0.88
		$\textrm {NPV}(0.9)$	0.417	0.002	0.016	0.015	0.90	0.49	0.002	0.015	0.014	0.89
Independent	10%	$\textrm {PPV}(0.1)$	0.583	−0.003	0.016	0.014	0.89	0.51	−0.003	0.016	0.015	0.89
		$\textrm {PPV}(0.5)$	0.583	−0.003	0.019	0.017	0.89	0.51	−0.003	0.02	0.018	0.88
		$\textrm {PPV}(0.9)$	0.583	−0.004	0.031	0.029	0.88	0.51	−0.002	0.031	0.027	0.89
		$\textrm {NPV}(0.1)$	0.417	0.003	0.031	0.029	0.87	0.49	0.006	0.031	0.028	0.88
		$\textrm {NPV}(0.5)$	0.417	0.003	0.022	0.018	0.88	0.49	0.004	0.021	0.019	0.89
		$\textrm {NPV}(.9)$	0.417	0.002	0.016	0.014	0.87	0.49	0.002	0.016	0.015	0.90
	30%	$\textrm {PPV}(.1)$	0.583	−0.003	0.017	0.015	0.89	0.51	−0.003	0.017	0.016	0.87
		$\textrm {PPV}(0.5)$	0.583	−0.003	0.020	0.018	0.88	0.51	−0.003	0.020	0.018	0.87
		$\textrm {PPV}(0.9)$	0.583	−0.002	0.032	0.028	0.89	0.51	−0.004	0.031	0.029	0.89
		$\textrm {NPV}(0.1)$	0.417	0.006	0.033	0.028	0.88	0.49	0.002	0.031	0.029	0.88
		$\textrm {NPV}(0.5)$	0.417	0.004	0.023	0.022	0.89	0.49	0.002	0.022	0.021	0.88
		$\textrm {NPV}(0.9)$	0.417	0.002	0.017	0.016	0.90	0.49	0.001	0.016	0.015	0.87
Covariate-dependent	35%	$\textrm {PPV}(0.1)$	0.583	−0.005	0.019	0.016	0.87	0.51	−0.004	0.018	0.016	0.86
		$\textrm {PPV}(0.5)$	0.583	−0.003	0.022	0.019	0.87	0.51	−0.005	0.019	0.018	0.87
		$\textrm {PPV}(0.9)$	0.583	−0.004	0.034	0.030	0.88	0.51	−0.004	0.031	0.029	0.89
		$\textrm {NPV}(0.1)$	0.417	0.008	0.034	0.029	0.88	0.49	0.002	0.033	0.028	0.87
		$\textrm {NPV}(0.5)$	0.417	0.006	0.024	0.021	0.89	0.49	0.003	0.02s	0.021	0.88
		$\textrm {NPV}(0.9)$	0.417	0.005	0.017	0.018	0.89	0.49	0.001	0.018	0.017	0.88

**TABLE 5:** Simulation results for the estimation of $\textrm {AUC}^{(r)}(t)$ of a baseline marker as a function of $v$ in baseline hazard, $\Lambda _{0}(t)=vt$, probability of censoring, Pr(C); and the degree of heterogeneity, $\theta$ and sample size $n$. The value of $r$ is fixed at a value of 2. We consider $t=5$ which represents a study of length 5 time units. The $\textrm {AUC}^{(r)}(t)$ estimation were conducted using the proposed semiparametric approach. The performance measures are bias (B), average of bootstrap standard errors (SE), standard deviation (SD), coverage probability (CP) (nominal level is 0.9). To reduce the burden of notation, we have dropped the terms $r$ and $t$ from the notation of $\textrm {AUC}^{(r)}(t)$. Here, $\textrm {AUC}^{(r)}(t)$ is represented by $\textrm {AUC}$.

			$\theta$ = 0						$\theta$ = 0.5					$\theta$ = 1				
Censoring	Pr(C)	$v$	$n$	True	B	SE	SD	CP	True	B	SE	SD	CP	True	B	SE	SD	CP
Complete follow-up	0%	0.5	200	0.790	−0.008	0.013	0.014	0.89	0.705	0.010	0.023	0.024	0.88	0.665	0.005	0.024	0.025	0.91
			500	0.760	−0.009	0.015	0.014	0.88	0.691	0.012	0.011	0.024	0.87	0.652	0.004	0.022	0.020	0.90
			1000	0.750	−0.008	0.014	0.013	0.89	0.681	0.009	0.011	0.014	0.89	0.640	0.003	0.023	0.021	0.90
Independent	25%	1.0	200	0.805	−0.002	0.015	0.017	0.89	0.712	−0.003	0.021	0.022	0.89	0.664	−0.001	0.023	0.024	0.89
			500	0.741	−0.011	0.015	0.013	0.88	0.682	−0.009	0.016	0.014	0.89	0.647	−0.004	0.021	0.019	0.89
			1000	0.732	−0.005	0.015	0.013	0.88	0.678	−0.007	0.018	0.016	0.89	0.640	−0.001	0.019	0.018	0.89
Covariate-dependent	40%	1.4	200	0.805	−0.003	0.015	0.016	0.88	0.708	0.002	0.021	0.022	0.89	0.664	0.001	0.023	0.025	0.89
			500	0.741	−0.011	0.015	0.013	0.88	0.682	−0.009	0.016	0.014	0.89	0.647	−0.004	0.021	0.019	0.89
			1000	0.741	−0.011	0.015	0.013	0.88	0.682	−0.009	0.016	0.014	0.89	0.647	−0.004	0.021	0.019	0.89

## 5 AN APPLICATION TO CF

A randomized trial of human recombinant deoxyribonuclease I (rhDNase) to treat CF was conducted by Genentech, Inc. in 1992 to compare rhDNase to placebo. At enrollment, the patient’s FEV was measured, and then the patient was monitored for pulmonary exacerbations. Patients with CF have abnormal levels of FEV. The FEV value helps clinicians manage the occurrence of pulmonary exacerbation efficiently. The exacerbation was defined as an infection that required the use of intravenous antibiotics. The dataset contains 647 patients, and they had 0–5 episodes of pulmonary exacerbations during the trial. The primary endpoint was the time until the first pulmonary exacerbation; however, data on all exacerbations were collected for 169 days. We obtained the rhDNASE dataset with the R package survival.

Our exploratory analysis revealed that approximately 63 percent of patients did not experience any episodes of exacerbation by 169 days since the beginning of the trial. Only 11 percent of patients experienced at least 2 episodes, and the remainder experienced only one episode. Note that the rhDNASE dataset also contained information regarding treatment assignment (rhDNASE vs placebo). Since only 11 percent of patients experienced at least 2 episodes, further separation by treatment assignment would have reduced the effective number of patients. Hence, we merged the 2 arms for analysis.

In this analysis, our goals are (1) to evaluate the performance of FEV as a biomarker to separate patients with a higher number of episodes from patients with a lower number of episodes; (2) to estimate the patient’s risk of recurrent episodes given the FEV value. The first goal is assessed using the estimated $\text{ROC}^{(r)}(p_{0},t)$ and $\text{AUC}^{(r)}(t)$ parameters. The latter goal is quantified using $\text{PPV}^{(r)}(v,t)$ and $\text{NPV}^{(r)}(v,t)$ curves.

Note that a lower FEV value means that the lung disease is worse (eg, Liou et al., 2001; de Boer et al., 2011; Wojewodka et al., 2014). Recall that the proposed methods assumed a higher marker value is more indicative of severe disease; therefore, we use the negative value of FEV as a marker for recurrent exacerbations. The conversion to the negative FEV will confirm that the risk of exacerbation is higher with a higher value of negative FEV. We name the negative value of FEV as “reversed FEV.” In addition, we consider the value of $r$ as 2 and 3. For example, in the case of $r=2$, the high-risk group will contain patients with 2 or more episodes of exacerbations by 169 days of follow-up, while low-risk groups will include patients with a maximum of one episode during the same time window.

**FIGURE 1:**
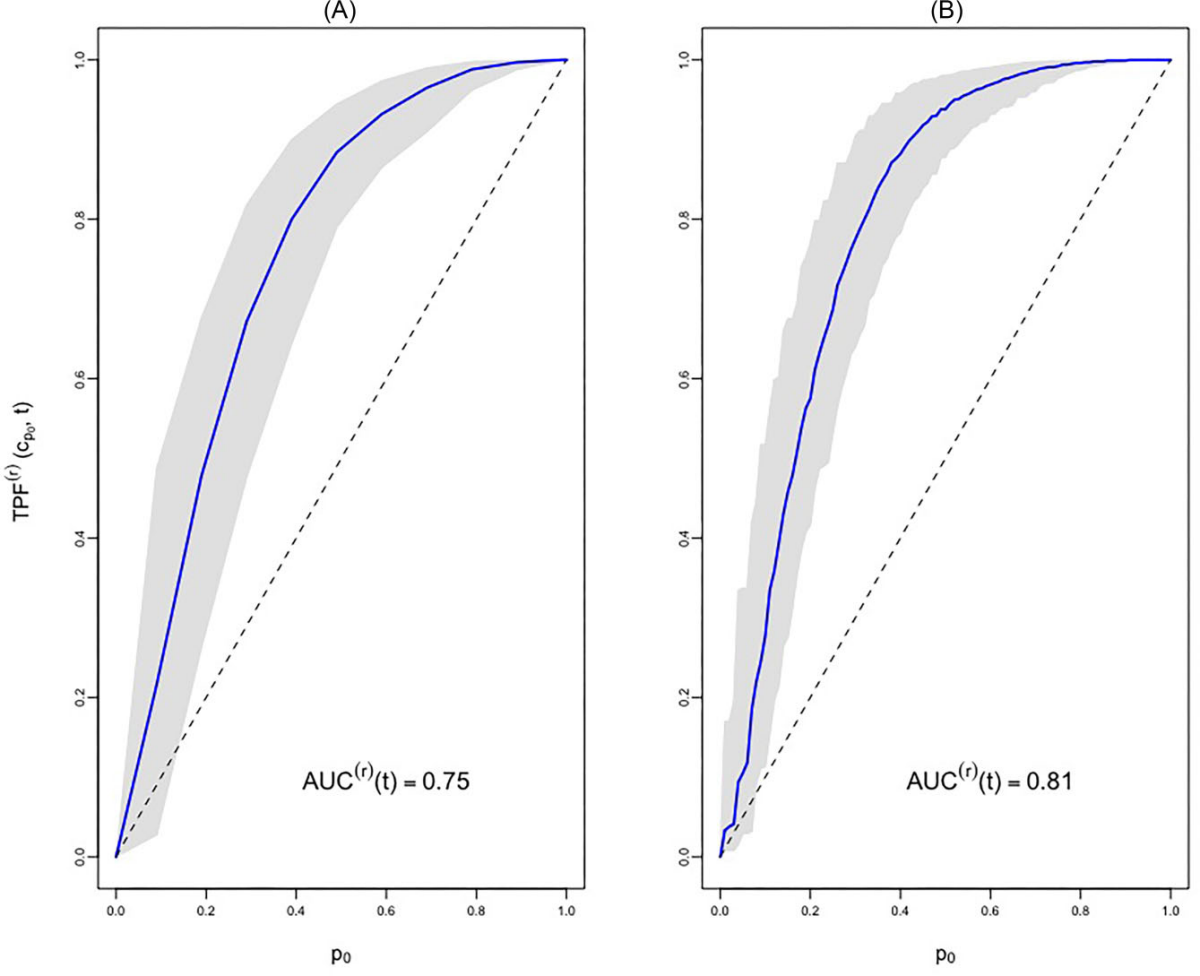
The receiver operating characteristic, $\text{ROC}^{(r)}(p_{0},t)$, curve is based on the semiparametric estimation procedure applied to the CF data. Here, $\text{TPF}^{(r)}(c_{p_{0}},t)$ represents the true positive probability, where $c_{p_{0}}$ is the threshold that yields a false positive probability of $p_{0}$, that is, $\text{FPF}^{(r)}(c_{p_{0}},t)=p_{0}$. Therefore, the $\text{ROC}^{(r)}(p_{0},t)$ is $\text{TPF}^{(r)}(c_{p_{0}},t)$. The $\text{AUC}^{(r)}(t)$ represents the area under the $\text{ROC}^{(r)}(p_{0},t)$ curve. The diagonal dotted line corresponds to an uninformative marker with $\text{AUC}^{(r)}(t)=0.5$. The left $\text{ROC}^{(r)}(p_{0},t)$ curve in (A) is based on $r=2$ and it quantifies, how well forced expiratory volume measured at baseline discriminates high-frequency patients (more than one pulmonary exacerbation) by 169 days of follow-up from low-frequency patients (maximum one pulmonary exacerbation) during the same time window. The right $\text{ROC}^{(r)}(p_{0},t)$ curve in (B) is based on $r=3$.

The ROC curves in Figures 1A and B illustrate the discriminative ability of FEV by the means of estimated ROC curves for $r=2$ and 3, respectively. Figure 1A shows that the estimated value of AUC is 0.75 for $r=2$. This is the probability that a randomly selected pair of high-risk (2 or more exacerbations) and low-risk (at most one exacerbation) patients are correctly ordered by their respective FEV values. When we change the “grouping criterion,” that is $r=3$, this probability goes up to 0.81 (Figure 1B), implying that the FEV score is more accurate in separating the patients with at least 3 episodes from the patients with at most of 2 episodes.

**FIGURE 2:**
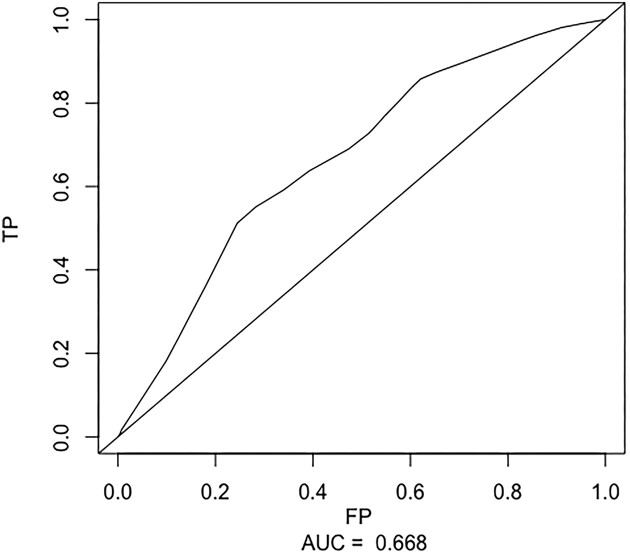
The receiver operating characteristics curve is applied to the cystic fibrosis data after terminating the follow-up for a patient when the first event occurs or (if it has not yet) when the study ends. The estimation is based on the semiparametric estimation procedure in Heagerty et al. (2000). The area under this curve AUC quantifies, how well forced expiratory volume measured at baseline discriminates patients with a single episode of pulmonary exacerbation by 169 days of follow-up from patients who have not experienced any episode of pulmonary exacerbation during the same time window. The diagonal dotted line corresponds to an uninformative marker with $\text{AUC}=0.5$.

For comparison purposes, we also carried out a time-to-first event analysis, with the end-point re-defined as the time to first pulmonary exacerbation. The result is provided in Figure 2. The area under this curve AUC quantifies how well FEV measured at baseline discriminates patients with a single episode of pulmonary exacerbation by 169 days of follow-up from patients who have not experienced any episode of pulmonary exacerbation during the same time window. We observed that the AUC decreased when only the time-to-first-pulmonary exacerbation was used (AUC = 0.66 for time-to-first-pulmonary exacerbation analysis and AUC = 0.75 for $r=2$). This stems from the use of recurrent event data, which enables a longer common observation period for risk comparisons. As a result, the corresponding model’s discriminatory power is increased.

**FIGURE 3:**
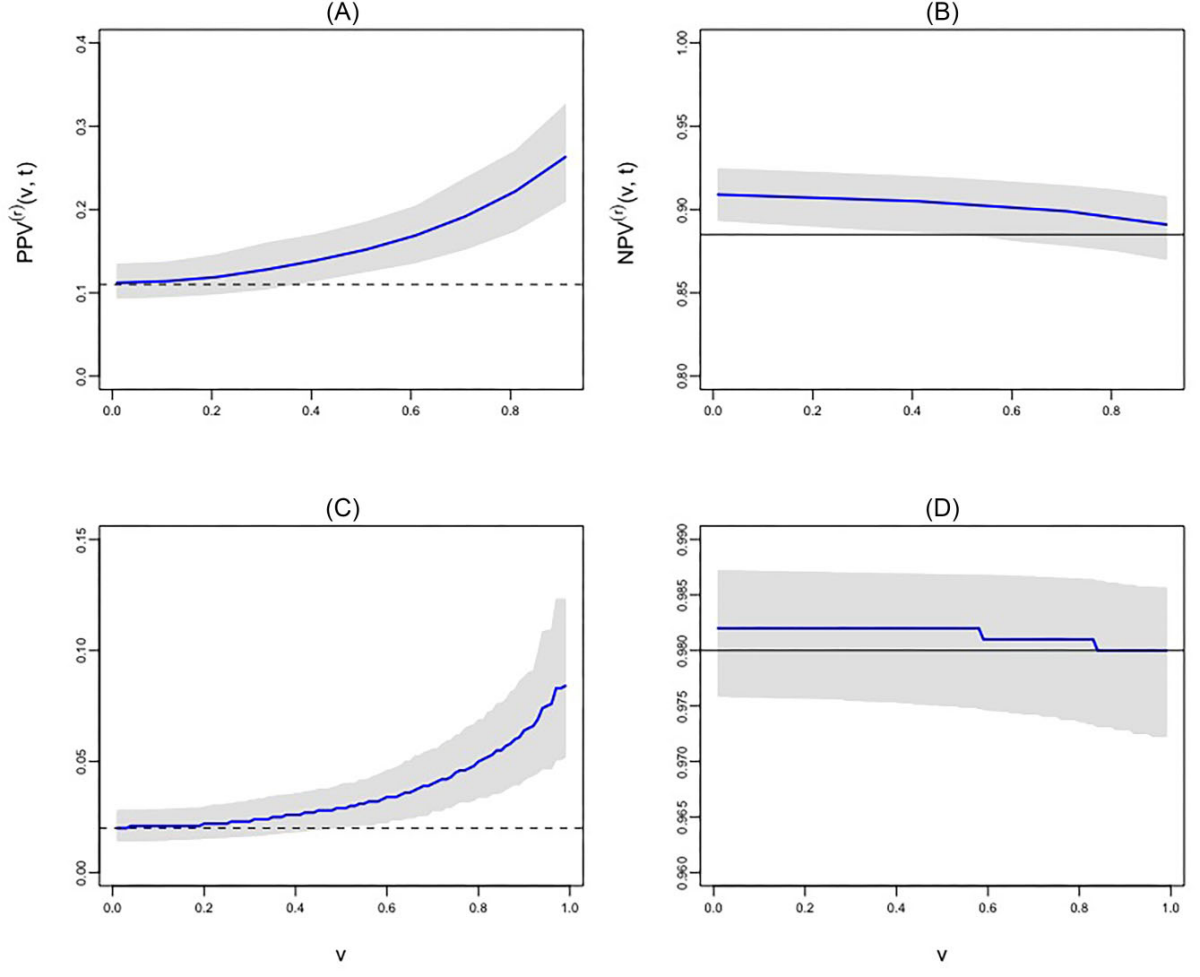
The positive predictive value, $\text{PPV}^{(r)}(v,t)$, and the negative predictive value, $\text{NPV}^{(r)}(v,t)$, curves are based on the semiparametric estimation procedure applied to the cystic fibrosis (CF) data. Here, $v$ refers to the fraction of patients with a positive CF result, when a positive result is defined by exceeding the threshold corresponding to the $v$th percentile of the marker in the population. The curves in the top row (ie, A and B) are based on $r=2$, and the curves in the bottom row are based on $r=3$. For example, in the case of $r=2$, the high-risk group contains patients with more than one exacerbation episode by 169 days of follow-up, while the low-risk group includes patients with a maximum of one episode during the same time window. Note that in plots (A) and (C), the dotted lines represent $\text{Pr}(N(t)\ge r)$ when $r$ = 2 and 3, respectively. In plots (B) and (D), the black lines represent $\text{Pr}(N(t)< r)$ when $r$ = 2 and 3, respectively.

Figure 3 displays $\text{PPV}^{(r)}(.)$ curves and $\text{NPV}^{(r)}(.)$ curves for $r=2,3$, respectively. Figure 3A illustrates that starting from the point $\text{Pr}(N(t)\ge 2)$ = 0.11, the PPV curve increases steeply, an indication that the FEV score is informative for identifying patients at greater risk of having at least 2 episodes of exacerbations by 169 days of follow-up. For example, at $v=0.5$ the corresponding $\hat{\text{PPV}}^{(r=2)}(v=0.5)=0.13,$ whereas $\hat{\text{PPV}}^{(r=2)}(v=0.95)=0.23$ at $v=0.95$. In other words, if the “reversed FEV” were used to refer patients for a novel therapy, among the patients whose reversed scores were in the top 5% of the population, on average 23% would have at least 2 episodes by 169 days of follow-up; however, among those patients whose scores were in the top 50% of the population, on average only 13% would have at least 2 episodes by 169 days of follow-up. Such information may be helpful for clinicians in determining which patients with a high-risk of recurrent episodes are eligible for more aggressive therapy.

If a PPV value $p$ by $t$, that is, $\hat{\text{PPV}}^{(r)}(v,t)=p$ was considered for clinical decision-making within time window [0, $t$], what percentage of the population, $v$, would be selected based on the “reversed FEV” We address this question by studying $v=[\hat{\text{PPV}}^{(r)}]^{-1}(p,t)$. For example, if the aim is to identify that 20% of patients have at least 2 recurrences by 169 days of follow-up, then from Figure 3A, we calculate that approximately 20% [ie, 1 − $v$ = 1 – 0.80] of the population must have positive result with the “reversed FEV.” In other words, we choose the 80th percentile of the “reversed FEV” as the threshold for defining positivity.

## 6 DISCUSSION

The performance of a prognostic marker should be rigorously evaluated before clinicians adopt it for use in routine clinical practice. In this manuscript, we present statistical methods for characterizing time-dependent accuracy measures of a baseline prognostic marker when the event of interest is recurrent. In the last several years, there has been substantial research on how to statistically evaluate markers that could be used for estimating the probability of disease onset or progression. However, little attention has been provided in developing accuracy measures to evaluate a marker’s prognostic ability for recurrent event time data. This research gap motivated us to develop accuracy measures for characterizing the prognostic accuracy in the recurrent events context.

To incorporate recurrent events, we give a definition of accuracy measures based on a counting process. These measures do not take into account the time to the events or the time between events directly, they only use the number of events within a certain time window. Based on this, we define 2 groups of high- and low-risk patients. Those who experience at least *r* events within a period are classified as “high-risk” patients, and those who have less than *r* events are classified as “low-risk” patients. Our proposed measures, for example, TPF, FPF, and ROC, evaluate a marker’s discriminative ability to separate the 2 groups. The area under the proposed ROC curve can be interpreted as the fraction of high-risk and low-risk pairs within $[0,\tau ]$, where the marker value for the high-risk patient is higher than the marker value for the low-risk patient. The measure reflects a marker’s ability to discriminate patients with respect to recurrent event risk. These accuracy measures will help the clinician identify those at high-risk for future recurrences from those who are not. In addition, identifying such prognostic markers to select patients for inclusion in randomized clinical trials could potentially result in more targeted studies and reduce the number of patients to recruit.

In modern evidence-based medicine, clinicians and individual patients are mostly concerned with risk prediction. However, TPF, FPF measures, and ROC curve are not useful when dealing with individual patient’s risk prediction. Thus, we have developed measures, for example, PPV and NPV curves, to assess the subject’s risk of recurrent episodes given the value of a marker.

Instead of using raw marker values in the *x*-axis of the PPV curve, we have used the fraction of patients result in positive. A “positive” result is defined by exceeding the threshold corresponding to the population’s $v$th percentile of a marker. The consideration of $v$ in the *x*-axis of the PPV curve is appealing; since it provides a meaningful way to find the threshold for test positivity. Also, this curve provides a common scale for comparing multiple markers measured on different scales.

The proposed estimators are based on a semiparametric frailty model that accounts for the informativeness of the marker and unobserved heterogeneity among patients on the rate of event occurrence. We assume a Gamma frailty distribution to characterize unobserved heterogeneity. The proposed estimators performed well in simulation studies in terms of bias and coverage. Additionally, we investigate the robustness of the proposed estimators under frailty model misspecification. The results indicate negligible bias under frailty model misspecification. Our procedures are simple yet meaningful for clinical practice and flexible in their assumptions about the underlying model.

The performance of this accuracy measure in comparing multiple markers is uncertain, given that the simulation and data analysis focus solely on a single marker. To validly compare multiple markers based on the proposed estimation, it’s essential to simultaneously validate the assumption of models for the rate function of the markers in Equations (9) and (10). If simultaneous validation is not guaranteed, then comparing multiple markers remains uncertain. This warrants further investigation.

In addition to that, our proposed approaches are not applicable when the data have missing information. However, following a typical imputation method, our approach could be applied to the imputed data. Details of the inference and sensitivity to the imputation methods are yet to be explored. In addition, in our setting, we do not consider time-varying markers. This is to be explored in the future.

## Supplementary Material

ujae150_Supplemental_Files The Web Appendix, Tables, and Figures, and data and R programming code referenced in Sections 3, 4 and 5 are available with this paper at the Biometrics website on Oxford Academic. The R programming code is available at https://github.com/rajibdey2024/recurrent.

## Data Availability

The data that support the findings in this paper were accessed from the rhDNASE dataset with the R package survival. It is available at https://cran.r-project.org/web/packages/survival/.

## APPENDIX A

### A.1 Asymptotic properties of the estimators

We first study the asymptotic properties of the proposed accuracy estimators. The key step to our theoretical development is the establishment of theory for the quantity ${f}_{j}(t,m)$. As shown in the Equation (12), the quantity ${f}_{j}(t,m)$ depends on $\Theta$. Let $\mathbb {M}$ be the support of the marker $M$.

## APPENDIX B PROOF OF LEMMA 1

In the view of Theorem 3 stated on page 202 of Parner (1998), $\sqrt{n}\, (\hat{\Theta }-{\Theta })$ converges to a Gaussian process with mean zero. The proof is provided on page 202 of Parner (1998). Thus, $\sqrt{n}\, (\hat{f}_{j}(t,m)-{f}_{j}(t,m))$, as a functional differentiable with respect to $\Theta$, converges to a Gaussian process on $\mathbb {M}$ by the functional delta method stated in Chapter 3.9 of van der Vaart and Wellner (1996).

## APPENDIX C PROOF OF THEOREM 1

Since ${\text{TPF}}^{(r)}(c,t)$ is differentiable as a composite functional of $\Theta$, by the functional delta method stated in Chapter 3.9 of van der Vaart and Wellner (1996), $\sqrt{n}\, (\hat{\text{TPF}}^{(r)}(c,t)-{\text{TPF}}^{(r)}(c,t))$ converges to a Gaussian process ${\bf T_{G}(m;t)}$ with mean zero on $\mathbb {M}$. To derive the asymptotic covariance, we can write

\begin{eqnarray*}
\sqrt{n}\, (\hat{\text{TPF}}^{(r)}(c,t)-{\text{TPF}}^{(r)}(c,t))=R_{1}+R_{2},
\end{eqnarray*}

where

\begin{eqnarray*}
R_{1} &=& \frac{n^{-1/2}\, \sum _{j=r}^{K} \int _{c}^\infty \hat{f}_{j}(t,m)\, d\hat{G}(m) }{n^{-1/2}\, \sum _{j=r}^{K} \int _{-\infty }^\infty \hat{f}_{j}(t,m)\, d\hat{G}(m)}\\
&&-\,\frac{n^{-1/2}\, \sum _{j=r}^{K} \int _{c}^\infty \hat{f}_{j}(t,m)\, d\hat{G}(m) }{n^{-1/2}\, \sum _{j=r}^{K} \int _{-\infty }^\infty {f}_{j}(t,m)\, d{G}(m)},
\end{eqnarray*}

\begin{eqnarray*}
R_{2} &=& \frac{n^{-1/2}\, \sum _{j=r}^{K} \int _{c}^\infty \hat{f}_{j}(t,m)\, d\hat{G}(m) }{n^{-1/2}\, \sum _{j=r}^{K} \int _{-\infty }^\infty {f}_{j}(t,m)\, d{G}(m)}\\
&&-\,\frac{n^{-1/2}\, \sum _{j=r}^{K} \int _{c}^\infty {f}_{j}(t,m)\, d{G}(m) }{n^{-1/2}\, \sum _{j=r}^{K} \int _{-\infty }^\infty {f}_{j}(t,m)\, d{G}(m)}.
\end{eqnarray*}

Using the functional Taylor expansion, with some algebra, we can show that

\begin{eqnarray*}
R_{1} &=& \frac{n^{-1/2}\, \sum _{j=r}^{K} \int _{c}^\infty \hat{f}_{j}(t,m)\, d\hat{G}(m) }{n^{-1/2}\, \sum _{j=r}^{K} \int _{-\infty }^\infty \hat{f}_{j}(t,m)\, d\hat{G}(m)} \\
&& \times\, \left(1-\frac{n^{-1/2}\, \sum _{j=r}^{K} \int _{-\infty }^\infty \hat{f}_{j}(t,m)\, d\hat{G}(m) }{n^{-1/2}\, \sum _{j=r}^{K} \int _{-\infty }^\infty {f}_{j}(t,m)\, d{G}(m)}\right),\\
&=& -n^{-1/2}\, \, {\text{TPF}}^{(r)}(c,t)\left\lbrace \sum _{j=r}^{K} \int _{-\infty }^\infty {f}_{j}(t,m)\, d{G}(m)\right\rbrace ^{-1} \\
&& \times \, \left[\int _{-\infty }^\infty \sum _{j=r}^{K} (\hat{f}_{j}(t,m)-{f}_{j}(t,m))\, d\hat{G}(m)\right] +o_{p}(1).
\end{eqnarray*}

Similarly, $R_{2}$ can be written as

\begin{eqnarray*}
R_{2} &=& n^{-1/2}\, \left\lbrace \sum _{j=r}^{K} \int _{-\infty }^\infty {f}_{j}(t,m)\, d{G}(m)\right\rbrace ^{-1} \\
&&\times\,\left[\int _{c}^\infty \sum _{j=r}^{K} (\hat{f}_{j}(t,m)-{f}_{j}(t,m))\, d\hat{G}(m)\right] +o_{p}(1).
\end{eqnarray*}

Hence,

\begin{eqnarray*}
\sqrt{n}\, (\hat{\text{TPF}}^{(r)}(c,t)-{\text{TPF}}^{(r)}(c,t))\\
= n^{1/2}\, \frac{1}{n}\sum _{i=1}^{n} \phi _{i}(m;t)+o_{p}(1),
\end{eqnarray*}

where

\begin{eqnarray*}
\phi _{i}(m,t) &=& \left\lbrace \sum _{j=r}^{K} \int _{-\infty }^\infty {f}_{j}(t,m)\, d{G}(m)\right\rbrace ^{-1} \\
&& \times \, \left [\int _{c}^\infty \sum _{j=r}^{K} (\hat{f}_{j}(t,m_{i})-{f}_{j}(t,m))\, d\hat{G}(m)\right] \\
&&-\, {\text{TPF}}^{(r)}(c,t)\, \left \lbrace \sum _{j=r}^{K} \int _{-\infty }^\infty {f}_{j}(t,m)\, d{G}(m)\right\rbrace ^{-1} \\ && \times \, \left [\int _{-\infty }^\infty \sum _{j=r}^{K} (\hat{f}_{j}(t,m_{i})-{f}_{j}(t,m))\, d\hat{G}(m)\right].
\end{eqnarray*}

Thus

(C.1)
\begin{eqnarray*}
\textrm {cov}\lbrace {\bf T_{G}(m_{1};t)}, {\bf T_{G}(m_{2};t)}\rbrace = \textrm {cov}\lbrace \phi _{{\it i}}(\bf {m_{1}};\bf {t}), \phi _{{\it i}}(\bf {m_{2}};\bf {t})\rbrace \\
\end{eqnarray*}

In practice, it is difficult to estimate $\textrm {cov}\lbrace \phi _{{\it i}}({\bf m_{1}};\bf {t}), \phi _{{\it i}}(\bf {m_{2}};\bf {t})\rbrace$, since it contains the density functions. Alternatively, we may compute the SEs and confidence bands using the bootstrap method.

Similarly, we can show that $\sqrt{n}\, (\hat{\text{FPF}}^{(r)}(c,t)-{\text{FPF}}^{(r)}(c,t))$ converges to a mean zero Gaussian process ${\bf T_{1G}(m;t)}$ on $\mathbb {M}$. To derive the asymptotic covariance, we can write

\begin{eqnarray*}
\sqrt{n}\, (\hat{\text{FPF}}^{(r)}(c,t)-{\text{FPF}}^{(r)}(c,t))=D_{1}+D_{2},
\end{eqnarray*}

where

\begin{eqnarray*}
D_{1} &=& \displaystyle\frac{n^{-1/2}\, \sum _{j=0}^{r-1} \int _{c}^\infty \hat{f}_{j}(t,m)\, d\hat{G}(m) }{n^{-1/2}\, \sum _{j=0}^{r-1} \int _{-\infty }^\infty \hat{f}_{j}(t,m)\, d\hat{G}(m)} \\
&&-\displaystyle\frac{n^{-1/2}\, \sum _{j=0}^{r-1} \int _{c}^\infty \hat{f}_{j}(t,m)\, d\hat{G}(m) }{n^{-1/2}\, \sum _{j=0}^{r-1} \int _{-\infty }^\infty {f}_{j}(t,m)\, d{G}(m)},
\end{eqnarray*}

\begin{eqnarray*}
D_{2} &=& \frac{n^{-1/2}\, \sum _{j=0}^{r-1} \int _{c}^\infty \hat{f}_{j}(t,m)\, d\hat{G}(m) }{n^{-1/2}\, \sum _{j=0}^{r-1} \int _{-\infty }^\infty {f}_{j}(t,m)\, d{G}(m)}\\
&&-\,\frac{n^{-1/2}\, \sum _{j=0}^{r-1} \int _{c}^\infty {f}_{j}(t,m)\, d{G}(m) }{n^{-1/2}\, \sum _{j=0}^{r-1} \int _{-\infty }^\infty {f}_{j}(t,m)\, d{G}(m)}.
\end{eqnarray*}

Using the functional Taylor expansion, with some algebra, we can show that

\begin{eqnarray*}
D_{1} &=& -n^{-1/2}\, \, {\text{FPF}}^{(r)}(c,t)\left\lbrace \sum _{j=0}^{r-1} \int _{-\infty }^\infty {f}_{j}(t,m)\, d{G}(m)\right\rbrace ^{-1}\, \\
&&\times\, \left[\int _{-\infty }^\infty \sum _{j=0}^{r-1} (\hat{f}_{j}(t,m)-{f}_{j}(t,m))d{G}(m)\right] +o_{p}(1).
\end{eqnarray*}

Similarly, $D_{2}$ can be written as

\begin{eqnarray*}
D_{2} &=& n^{-1/2}\left \lbrace \sum _{j=0}^{r-1} \int _{-\infty }^\infty {f}_{j}(t,m)\, d{G}(m)\right\rbrace ^{-1}\\
&&\times\, \left[\int _{c}^\infty \sum _{j=0}^{r-1} (\hat{f}_{j}(t,m)-{f}_{j}(t,m))\, d{G}(m)\right] +o_{p}(1).
\end{eqnarray*}

Hence,

\begin{eqnarray*}
\sqrt{n}\, (\hat{\text{FPF}}^{(r)}(c,t)-{\text{FPF}}^{(r)}(c,t))\\
\quad = n^{1/2}\, \frac{1}{n}\sum _{i=1}^{n} \psi _{i}(m,t)+o_{p}(1),
\end{eqnarray*}

where

\begin{eqnarray*}
\psi _{i}(m,t) &=& \left\lbrace \sum _{j=0}^{r-1} \int _{-\infty }^\infty {f}_{j}(t,m)\, d{G}(m)\right\rbrace ^{-1} \\
&& \times \,\left [\int _{c}^\infty \sum _{j=0}^{r-1} (\hat{f}_{j}(t,m_{i})-{f}_{j}(t,m))\, d{G}(m)\right] \\
&&-\,{\text{FPF}}^{(r)}(c,t)\left \lbrace \sum _{j=0}^{r-1} \int _{-\infty }^\infty {f}_{j}(t,m)\, d{G}(m)\right\rbrace ^{-1} \\ && \times \, \left [\int _{-\infty }^\infty \sum _{j=0}^{r-1} (\hat{f}_{j}(t,m_{i})-{f}_{j}(t,m))\, d{G}(m)\right].
\end{eqnarray*}

Thus

(C.2)
\begin{eqnarray*}
\textrm {cov}\lbrace {\bf T_{1G}(m_{1};t)}, {\bf T_{1G}(m_{2};t)}\rbrace = \textrm {cov}\lbrace \psi _{{\it i}}(\bf {m_{1}};\bf {t}), \psi _{{\it i}}(\bf {m_{2}};\bf {t})\rbrace. \\
\end{eqnarray*}

## APPENDIX D PROOF OF THEOREM 2

*Proof:*. Since ${\text{PPV}}^{(r)}(v,t)$ is differentiable as a composite functional of $\Theta$, by the functional delta method, $\sqrt{n}\, (\hat{\text{PPV}}^{(r)}(v,t)-{\text{PPV}}^{(r)}(v,t))$ converges to a Gaussian process with mean zero on $\mathbb {M}$. Similarly, the asymptotic covariance for this process can be derived using the functional Taylor expansion, we have

(D.1)
\begin{eqnarray*}
\sqrt{n}\, (\hat{\text{PPV}}^{(r)}(v,t)-{\text{PPV}}^{(r)}(v,t)) &=& \sqrt{n}\, (1-v)^{-1}\, \times \, \sum _{j=r}^{K}\, \Bigg[\int _{G^{-1}(v)}^\infty ( \hat{f}_{j}(t,m)-{f}_{j}(t,m))\, dG(m) \\
&&+\,\int _{G^{-1}(v)}^\infty {f}_{j}(t,m))\, d(\hat{G}(m)-G(m))\Bigg]+o_{p}(1) .
\end{eqnarray*}

Similarly, ${\text{NPV}}^{(r)}(v,t)$ is differentiable as a composite functional of $\Theta$, by the functional delta method, $\sqrt{n}\, (\hat{\text{NPV}}^{(r)}(v,t)-{\text{NPV}}^{(r)}(v,t))$ converges to a Gaussian process with mean zero on $\mathbb {M}$. Similarly, the asymptotic covariance for this process can be derived using the functional Taylor expansion, we have

(D.2)
\begin{eqnarray*}
\sqrt{n}\, (\hat{\text{NPV}}^{(r)}(v,t)-{\text{NPV}}^{(r)}(v,t)) &=& \sqrt{n}\, v^{-1}\, \times \, \sum _{j=r}^{K}\, \Bigg[\int _{-\infty }^{G^{-1}(v)}( \hat{f}_{j}(t,m)-{f}_{j}(t,m))\, dG(m)\\
&&+\,\int _{-\infty }^{G^{-1}(v)} {f}_{j}(t,m))\, d(\hat{G}(m)-G(m))\Bigg]+o_{p}(1).
\end{eqnarray*}

## APPENDIX E PROOF OF THEOREM 3

In the light of the proof of Corollary 3 of Amico et al. (2021), we can write

(E.1)
\begin{eqnarray*}
(\hat{\textrm {ROC}}^{(r)}(.\, ,t)-{\textrm {ROC}}^{(r)}(.\, ,t)):= L_1(.;t)+L_2(.;t), \\
\end{eqnarray*}

where

\begin{eqnarray*}
L_2(.;t) &=& \! \text{dTPF}^{(r)}\lbrace (1-\text{FPF}^{(r)})^{-1}(.,t)\rbrace \lbrace (1-\hat{\text{TPF}}^{(r)})^{-1}(.,t) \\
&& - \, (1-\text{TPF}^{(r)})^{-1}(.,t)\rbrace +o_{p}(1)\\
&=& \frac{\text{dTPF}^{(r)}\lbrace (1-\text{FPF}^{(r)})^{-1}(.,t)\rbrace }{(1-\text{dFPF}^{(r)})\lbrace (1-\text{FPF}^{(r)})^{-1}(.,t)\rbrace }\times n^{-1} \\
&& \times \sum _{i=1}^{n}\psi _{i}(m,(1-\text{FPF}^{(r)})^{-1}(.,t))+o_{p}(1).
\end{eqnarray*}

and

\begin{eqnarray*}
L_1(.;t) &=& \hat{\text{TPF}^{(r)}}\lbrace (1-\text{FPF}^{(r)})^{-1}(.,t)\rbrace \\
&& - \, \text{TPF}^{(r)}\lbrace (1-\text{FPF}^{(r)})^{-1}(.,t)\rbrace +o_{p}(1)\\
&=& n^{-1}\times \sum _{i=1}^{n}\phi _{i}(m,(1-\text{FPF}^{(r)})^{-1}(.,t))+o_{p}(1).
\end{eqnarray*}

Therefore, from Equation (E.1), we can write

(E.2)
\begin{eqnarray*}
(\hat{\textrm {ROC}}^{(r)}(.\, ,t)-{\textrm {ROC}}^{(r)}(.\, ,t))\\
= n^{-1} \times \sum _{i=1}^{n} \eta _{i}(m;t)+o_{p}(1),
\end{eqnarray*}

where

\begin{eqnarray*}
\eta _{i}(m;t) &=& \phi _{i}(m,(1-\text{FPF}^{(r)})^{-1}(.,t))\\
&&+\,\frac{\text{dTPF}^{(r)}\lbrace (1-\text{FPF}^{(r)})^{-1}(.,t)\rbrace }{(1-\text{dFPF}^{(r)})\lbrace (1-\text{FPF}^{(r)})^{-1}(.,t)\rbrace } \\
&& \times\, \psi _{i}(m,(1-\text{FPF}^{(r)})^{-1}(.,t)).
\end{eqnarray*}

Note that, ${\textrm {ROC}}^{(r)}(.\, ;t)$ is a composite functional of ${f}_{j}(t,m);\, \, j\, \, \epsilon \, \, (0,2,\dots ,K)$, then by the functional delta method, $\sqrt{n}\, (\hat{\textrm {ROC}}^{(r)}(.\, ;t)-{\textrm {ROC}}^{(r)}(.\, ;t))$ converges to a Gaussian process ${\bf T_{2G}(m;t)}$ with mean zero and covariance function

(E.3)
\begin{eqnarray*}
\textrm {cov}\lbrace {\bf T_{2G}(m_{1};t)}, {\bf T_{2G}(m_{2};t)}\rbrace = \textrm {E}\lbrace \eta _{{\it i}}(\bf {m_{1}};\bf {t}), \eta _{{\it i}}(\bf {m_{2}};\bf {t})\rbrace . \\
\end{eqnarray*}
